# Stability and Bioaccessibility of Quercetin-Enriched Pickering Emulsion Gels Stabilized by Cellulose Nanocrystals Extracted from Rice Bran

**DOI:** 10.3390/polym16070868

**Published:** 2024-03-22

**Authors:** Guozhen Wang, Jin Li, Xiaoqin Yan, Yan Meng, Yanpeng Zhang, Xianhui Chang, Jie Cai, Shilin Liu, Wenping Ding

**Affiliations:** 1 Key Laboratory for Deep Processing of Major Grain and Oil, Ministry of Education, School of Food Science and Engineering, Wuhan Polytechnic University, Wuhan 430023, China; lupananran777@163.com (J.L.); yxq18728799861@163.com (X.Y.); ayzyp@126.com (Y.Z.); cxh5286@whpu.edu.cn (X.C.); caijievip@whpu.edu.cn (J.C.); 2 School of Pharmacy, Hubei University of Chinese Medicine, Wuhan 430065, China; yanmeng2016@126.com; 3 College of Food Science & Technology, Huazhong Agricultural University, Wuhan 430070, China

**Keywords:** rice bran, cellulose nanocrystals, Pickering emulsion, gel emulsification, quercetin, bioaccessibility

## Abstract

To investigate the optimal delivery system of quercetin, in this paper, cellulose nanocrystals (CNCs) extracted from rice bran were used to stabilize the Pickering emulsion and Pickering emulsion gels (PEGs) with quercetin. To compare the emulsion properties, stability, antioxidation activity, encapsulation rate, and bioaccessibility of the quercetin, four emulsions of CNC Pickering emulsion (C), CNC Pickering emulsion with quercetin (CQ), CNC Pickering gel emulsion (CG), and CNC Pickering gel emulsions with quercetin (CQG) were prepared. All four emulsions exhibited elastic gel network structure and good stability. The quercetin significantly reduced the particle size, increased the stability, and improved the antioxidant capacity of CQ and CQG. Compared to C and CG, the ABTS^+^ radical scavenging capacities of CQ and CQG were respectively enhanced by 46.92% and 3.59%. In addition, CQG had a higher encapsulation rate at 94.57% and higher bioaccessibility (16.17) compared to CQ. This study not only indicated that CNC from rice bran could be exploited as an excellent stabilization particle for Pickering emulsions, but also provided a highly stable and bioaccessible delivery system for water-insoluble functional active factors.

## 1. Introduction

In recent years, functional food factors have been considered an essential research direction to enhance the value of food products. Quercetin has shown antioxidant, anti-inflammatory, anticancer, and antibacterial effects in the study of beneficial bioactive functions [1]. However, quercetin lacks good water solubility and vital chemical stability, resulting in low bioaccessibility and limited applications in food and medicine [2]. To improve the bioavailability of quercetin and ensure its targeted delivery and absorption, the encapsulation of quercetin in an emulsion system has received extensive attention [3].

Pickering emulsion (PE) is an emerging delivery system in which the solid particles are distributed at the interface of two or more phases to form a dense interfacial film to stabilize the kinetic metastable emulsion system. Nowadays, there are more studies utilizing PE systems to deliver quercetin. Luo found that flavonoids could be adsorbed onto the surface of oil droplets in the form of water-insoluble particles that directly act as stabilizers for oil-in-water emulsions, thus facilitating delivery and absorption in the intestinal tract. However, he also emphasized that the formed emulsion droplets were relatively large due to the size of the flavonoid particles [4]. So, its practical significance remains to be seen.

To address this issue, attention has been drawn to PE stabilized by nanocrystals prepared from natural macromolecules such as cellulose. Cellulose nanocrystals (CNCs) have been proven to be outstanding emulsifiers for PE due to their high crystallinity, small size, large specific surface area, and the strong interactions of hydrogen bonding between CNC nanoparticles [5,6]. By constructing O/W PE, CNCs could be distributed on the surface of emulsion droplets and stable PE, encapsulating water-insoluble functional food factors. In addition, CNCs are widely sourced. They can be extracted from agricultural waste materials, including cork [7], rice husk [8,9], maize cob [10], pineapple leaves [11], garlic straw [12], wheat bran [13], and rice bran [14]. CNCs from celery stalk as quercetin scaffolds by a simple physical mixture was used for human holo-transferrin adsorption and digestion behaviors by Chamani [15]. Cellulose nanofiber could provide a promising nanoformulation candidate for quercetin by adjusting the solvent composition ratio to obtain cellulose nanofiber/quercetin nanoformulation with a high loading capacity of 78.91% and encapsulation efficiency of 88.77% [16]. However, there was no report about the nanoformulation of CNC/quercetin through a Pickering emulsion. 

In addition, Pickering emulsion gels (PEGs), gel-like Pickering emulsion systems, are also a new type of delivery system. They combine with the virtues of emulsions and gels, such as superior environmental stability, flexibility of structural adjustment, loading ability, and controlled release of bioactive substances. These systems can provide a critical platform medium for functional factors encapsulation and their slow release in food, material science, and pharmaceutics [17]. Wang prepared a quercetin emulsion gel with a bioavailability of 55.01% using whey protein isolate and hyaluronic acid by combining heating and treatment with CaCl_2_ [18]. Chen utilized gelatin to prepare PEGs encapsulating quercetin with a 97.2% encapsulation rate and fourfold increase in bioavailability [19]. Furthermore, Zhang also discovered that combining polysaccharides and proteins in food gel formulations is beneficial for enhancing bioactivity while overcoming technical challenges such as pH sensitivity and low gel properties [20]. Based on this, in order to increase the bioavailability of quercetin, we can select gelatin as the gelling agent for the preparation of gel emulsions.

In a word, in our paper, we focused on the encapsulation rate and bioaccessibility of quercetin by PEs and PEGs. Rice bran is rich in cellulose biomass [21]. In 2020 alone, 12.71 million tons of rice bran was produced in China. But unfortunately, cellulose, especially CNCs isolated from rice bran, is not well developed [22]. At first, in order to improve the utilization rate of rice bran, we aimed to prepare PEs and PEGs stabilized by CNCs extracted from rice bran. Then, to determine the most suitable delivery system, the differences between PEs and PEGs in improving the stability and biological activity of quercetin were compared. This study will provide new ideas for the highly valuable utilization of cellulose from rice bran and provide important theoretical and technical guidance for the delivery of quercetin and other functional food factors.

## 2. Experimental

### 2.1. Materials

Defatted rice bran was provided free of charge by Yihai Kerry Grain and Oil Industry Co., Ltd. (Wuhan, China). Corn oil was purchased from a local supermarket (Yihai Kerry Grain and Oil Industry Co., Ltd., Wuhan, China). Porcine pancreatic enzyme, α-Amylase, pancreatic enzyme, and calcofluor white were provided by Sigma-Aldrich (St. Louis, MO, USA). Sodium hydroxide, ethanol, hydrogen peroxide (Sinopharm Co., Ltd., Shanghai, China), and all the other reagents (Shanghai Yuanye Biotechnology Co., Ltd., Shanghai, China) were of analytical grade and used as received. 

### 2.2. Preparation of CNCs from Rice Bran 

The preparation of rice bran cellulose proceeded as follows: 50 g of defatted rice bran was dispersed in 500 mL of deionized water according to 1:10, and then, 0.25 g of α-amylase was added. The mixture was shaken for 40 min at 80 °C in the water bath and, then, centrifuged to take the precipitate and set aside. The above operation was repeated three times. The resulting precipitate was refluxed in 4% NaOH for 3 h at a ratio of 1:20, and the operation was repeated twice and washed thoroughly to neutral. Then, it was bleached using hydrogen peroxide solution (1.2 wt%) at 80 °C for 3 h. The bleaching was repeated three times, and the solid was washed to neutrality. Finally, the samples were freeze-dried (KLGH-18, Beijing Songyuan Huaxing Technology Development Co., Ltd., Beijing, China) to obtain white cellulose powder.

The preparation of rice bran CNCs proceeded as follows: the above rice bran cellulose was dispersed in 58% sulfuric acid and stirred at 45 °C for 3 h. After hydrolysis, deionized water was added to terminate the reaction. The residual solid was washed three times and then was dialyzed with deionized water to neutral. The obtained suspension was sonicated using a cell crusher (JY98-ⅢDN, Ningbo Xinzhi Bio-technology Co., Ningbo, China) with ultrasound power of 800 w and an ultrasonic time of 30 min. The resulting suspension was centrifuged to remove the larger particle sizes of cellulose to prepare rice bran CNCs [8,13].

### 2.3. Characterization of CNCs Isolated from Rice Bran

To detect the FTIR of CNCs isolated from rice bran, 1% of the samples was thoroughly mixed with KBr powder, and transparent flakes were obtained by pressing. The spectra were recorded on an FTIR spectrometer (Frontier, PerkinElmer Instruments Co., London, UK) at a resolution of 4 cm^−1^ with 64 scans. XRD patterns were measured using an X-ray diffractometer (Bruker D8 Advance, Brooke Technology Co., Ltd., Beijing, China) with Cu-Kα radiation (λ = 1.5406 Å) with scanning mode at a speed of 2°/min. The microstructure of the CNCs was examined by a transmission electron microscope (TEM, JEM-2000, Electronics Photonics Co., Tokyo, Japan) at an accelerating voltage of 80 kV.

### 2.4. Preparation of PE and PEGs

The preparation of CNC Pickering emulsion (named as C) proceeded as follows: 0.5% (wt%) of the CNC suspension prepared above was used as the aqueous phase. The aqueous phase and the oil phase (corn oil) were placed in a glass bottle with the ratio of 7:3. It was cut at 10,000 rpm for 3 min using a high-speed disperser (XHF-DY, Ningbo Xinzhi Biotechnology Co., Ningbo, China) and then homogenized for 5 min using a high-pressure homogenizer (AH-2010, ATS Engineering Inc., Shanghai, China) at a pressure of 20 Mpa. 

The preparation of CNC-Pickering gel emulsion (named as CG) proceeded as follows: Pickering emulsion and 10% gelatin solution were mixed with thorough stirring in a 40 °C water bath at a ratio of 5:1 [23]. 

The preparation of emulsion containing quercetin was similar to the above steps, with the difference that quercetin powder was added to corn oil and mixed using a magnetic stirrer until fully dissolved to form a new oil phase. The final concentration of quercetin in the emulsions was 0.1% (*w/w*). The CNC Pickering emulsion with quercetin was named CQ, and the CNC Pickering gel emulsion with quercetin was noted as CQG. All four emulsions mentioned above were stored at 4 °C [24]. 

### 2.5. Particle Size and Zeta Potential of the Emulsions

The particle size of the emulsion was determined using a laser particle-size meter (Mastersizer 3000, Malvern Instruments Co., Malvern, UK), and the zeta potential was determined using a particle-size meter (Malvern ZEN3600, Malvern Instruments Co., Malvern, UK).

### 2.6. Confocal Laser Scanning Microscopy of Emulsions

Confocal laser scanning microscopy (CLSM) images of the emulsions were obtained using an OLYMPUS Fluoview Fvloi confocal microscope (Japan Olympus Instrument Co., Ltd., Tokyo, Japan). Then, 500 μL of the emulsion was added to a 1.5 mL Eppendorf tube and mixed with 10 μL of Nile red (0.1% *w/v* in alcohol, excitation 514 nm, emission 539–648 nm) and 100 μL of calcofluor white (1.0% *w/v* in Milli-Q water, excitement 405 nm, emission 410–523 nm). The mixture was vortexed for 10 s and balanced for 10 min. After that, 10 μL of the mixture was dropped onto a concave slide. It was covered with a coverslip, and the images were observed on the computer [23].

### 2.7. Rheological Performance of the Emulsions 

The apparent viscosity, elastic module (G’), and viscous modulus (G’’) of the emulsions were determined at 25 °C using a Kinexus Pro+ multifunctional rotational rheometer (Malvern Instruments Ltd., Malvern, UK). A 60 mm diameter tapered plate with an adjusted gap of 0.05 mm was selected for the fixture. The rheological properties of the emulsions were measured at 25 °C. The linear viscoelastic range was measured with a strain scan (0.01–100%) at a fixed frequency of 10 rad/s. A dynamic frequency scan was then performed at 0.5% over a frequency range of 0.1 to 100 rad/s to determine the energy storage module (G’) and loss modulus (G’). The change in the viscosity of the emulsion as a function of the shear rate was measured by increasing the sear rate from 0.01 s^−1^ to 100 s^−1^. The apparent viscosity (ηa) was calculated using the device software and expressed as a function of the shear rate [25].

### 2.8. Stability of the Emulsions 

Emulsion stability was measured with TurBiscan Lab (France Formulaction Instruments Ltd., Toulouse, France). Then, 20 mL of the fresh emulsion was placed in a 43 mm high flat-bottomed glass cuvette, and the cuvette was scanned at 25 °C for 1 h. Emulsion destabilization was analyzed using backscattering (BS) curves for different sample heights. The sample with a height of 0 mm corresponds to the bottom of the cuvette. The Turboscan stability index (TSI) was used to determine the stability of the whole dispersion system [26,27].

### 2.9. Storage Stability of the Emulsions 

The freshly prepared emulsions were stored at 4 °C for 14 d, and the particle size and zeta potential of the emulsions were measured at 7-day intervals [28].

### 2.10. Oxidative Stability of Emulsions

To assess the oxidative stability of the emulsions, a fixed volume of fresh emulsion was transferred to glass tubes and stored in the dark at 50 °C for 15 d to allow for autoxidation. Lipid oxidation was determined by peroxide value (POV) and thiobarbituric acid reactive substances (TBARS) methods at 0, 3, 6, 9, 12, and 15 d, respectively. 

The POVs of different emulsions were measured using the method described by Thamonwan et al. [29]. First, 0.3 mL of the sample and 1.5 mL of isooctane/2-propanol mixture (3:1 *v*/*v*) solution were evenly mixed. These composite samples were centrifuged at 10,000 rpm and 25 °C for 5 min. Then, 0.2 mL of the supernatant was removed. Next, 2.8 mL of the methanol/1-butanol mixture (2:1, *v*/*v*), 15 μL of NH_4_SCN (3.94 M), and 15 μL of Fe^2+^ (fresh) were added. Finally, the reaction was conducted for 20 min in a dark environment. The absorbance at 510 nm was measured using a spectrophotometric microplate reader (EnSpire^®^ Multimode Plate reader, Perkin Elmer Management Co., Ltd., Shanghai, China). POVs were calculated using a standard hydrogen peroxide/isopropyl benzene curve (0–20 μM). 

The TBARS levels for different emulsions were measured according to the method described by Hu, Xie et al. [30]. In brief, 2 mL of TBA reagent (15% trichloroacetic acid (*w/v*) and 0.375% thiobarbituric acid (*w/v*) in 0.25 M HCl) was mixed with 1 mL of sample, boiled for 15 min, and cooled. Then, the samples were centrifuged at 10,000 rpm and 25 °C for 15 min to measure the absorbance of the supernatant at 532 nm. TBARS levels were obtained using a standard curve (0–20 μM 1, 1, 3, 3-tetra ethoxy propane, which reacts to form equal malondialdehyde molarity).

### 2.11. Antioxidation Activity (AA) of the Emulsions

ABTS (2,2′-azinobis-(3-ethylbenzthiazoline-6-sulphonate)) radical scavenging activity: ABTS^+^ was obtained by mixing 7 mM of aqueous ABTS^+^ solution with 2.45 mM of K_2_S_2_O_8_ solution at a ratio of 1:2 (V/V). The ABTS^+^ solution was stored in a dark environment for more than 16 h before use. The ABTS^+^ solution was further diluted with ethanol to produce an absorbance of 0.7 ± 0.02 at the wavelength of 734 nm. Then, 0.3 mL of the emulsion was mixed with 1.2 mL ABTS^+^ dilution for 20 min and then centrifuged at 12,000 rpm for 5 min. The supernatant was collected, and the absorbance was recorded at 734 nm [31]. The following equation was used to calculate the ability of the sample to remove ABTS^+^:

$$\mathrm{ABTS}\;\mathrm{radical}\;\mathrm{scavenging}\;\mathrm{activity}\;(\%)=\frac{A_{0}-(A_{2}-A_{1})}{A_{0}}\times 100$$

where A_0_ is the absorbance of deionized water and ABTS solution, A_1_ is the absorbance of the sample and ethanol, and A_2_ is the absorbance of the sample and ABTS solution.

For the DPPH radical scavenging activity: the emulsion was diluted 10 times with deionized water. Then, 1.2 mM of DPPH solution was prepared using ethanol, and 2 mL of DPPH–ethanol solution was added to 1 mL of the sample solution and reacted at room temperature in a dark environment for 30 min. The absorbance was measured at 517 nm [31]. The calculations were as follows:

$$\mathrm{DPPH}\;\mathrm{radical}\;\mathrm{scavenging}\;\mathrm{activity}\;(\%)=\frac{A_{0}-(A_{2}-A_{1})}{A_{0}}\times 100$$

where A_0_ is the absorbance of deionized water and DPPH solution, A_1_ is the absorbance of the sample and ethanol, and A_2_ is the absorbance of the sample and DPPH solution.

### 2.12. Encapsulation Rate and Bioaccessibility of the Quercetin

The emulsion-encapsulated quercetin was diluted 50 times with DMSO and centrifuged at 10,000 rpm for 15 min. The absorbance A_2_ was measured at 370 nm [32]. The encapsulation rate (EE) was calculated according to the following equation:

$$\mathrm{EE}\;(\%)=\frac{A_{1}-A_{2}}{A_{1}}\times 100$$

where A_1_ is the total amount of initially added quercetin.

Bioaccessibility was determined according to the following equation [33,34]:

$$\%\mathrm{bioaccessibility}=\frac{\mathrm{quercetin}\;\mathrm{content}\;\mathrm{in}\;\mathrm{the}\;\mathrm{micelle}\;\mathrm{phase}}{\mathrm{total}\;\mathrm{quercetin}\;\mathrm{content}\;\mathrm{in}\;\mathrm{the}\;\mathrm{formulations}}\times 100$$

## 3. Results and Discussion 

### 3.1. Characterization of CNCs Isolated from Rice Bran

**Figure 1:**
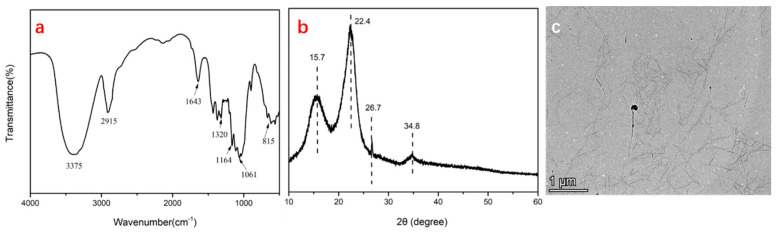
FTIR spectrum (**a**), XRD curve (**b**), and TEM image (**c**) of CNCs extracted from rice bran.

The chemical structure of CNCs isolated from rice bran was detected by FTIR and XRD. As shown in Figure 1a, the peaks at 3375 cm^−1^ and 2915 cm^−1^ were assigned to the stretching vibration of O-H and C-H in the CNCs. The absorption peak at 1643 cm^−1^ corresponded to the little water adsorbed in the samples. The frequency of C-O-C in the glucosidic bond was located at 1320 cm^−1^. Typical peaks at 1164 cm^−1^ and 1061 cm^−1^ were associated with the asymmetric tensile vibration of C-O-C and stretching vibrations of C-O-C. In particular, the peak that occurred at 815 cm^−1^ was related to the vibration of C-O-S in sulfite, which was formed during the hydrolysis of cellulose in sulfuric acid. In Figure 1b, the typical diffraction peaks of cellulose was observed at 2θ = 15.7°, 22.4°, and 34.8°, corresponding to the (110), (002), and (004) planes of cellulose I, respectively. A TEM photograph recorded the micromorphology of the CNCs in Figure 1c. Large amounts of vimineous and needlelike CNCs were well dispersed in the photo. Based on these results, pure cellulose nanocrystals were prepared.

### 3.2. Emulsion Particle Size, Zeta-Potential Analysis

The particle size and zeta potential of emulsions are essential indicators for evaluating the stability of emulsions. As shown in Table 1, the d_[4,3]_ and d_[3,2]_ particle sizes are respectively as follows: C, 4.59 and 2.93 μm; CQ, 3.17 and 2.24 μm; CG, 7.12 and 3.15 μm; CQG, 6.91 and 2.54 μm. This result indicated that the addition of quercetin significantly reduced the particle size due to the reduction in interfacial tension through the adsorption of quercetin at the interface. Similar to the literature [35], the addition of 0.60 wt% quercetin reduced the drop size of the Pickering double emulsions. The addition of gelatin to the Pickering emulsions to form gel-type emulsions increased the particle size of the emulsion. Zeta potential is another influencing factor that affects the stability of Pickering emulsions. The higher absolute value of zeta potential, the stronger the repulsive effect and the greater the ability to avoid aggregation of the dispersed-phase liquid to form stable Pickering emulsions [36,37]. The absolute values of the zeta potentials of the Pickering and gel emulsions were about 41 mV and 12 mV, respectively, which were similar to previous results [38].

### 3.3. Laser Confocal Microscopy Analysis of Emulsions

**Figure 2:**
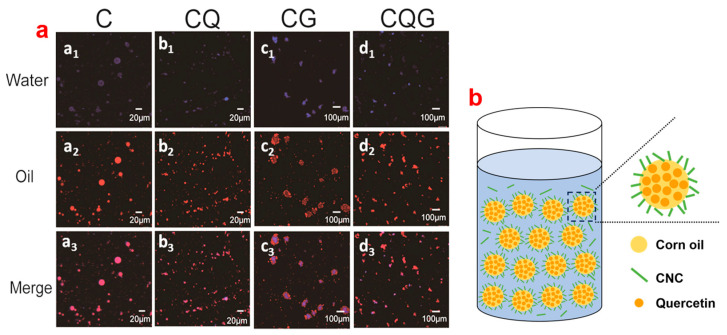
Image of emulsion laser confocal microscope (**a**) and the schematic diagram of emulsions (**b**).

The results of laser confocal microscopy revealed the effect of CNCs on the micro-structure of emulsions. As shown in Figure 2a, the blue calcofluor white represented CNC solid particles and the Nile-red-labeled represented oil phase. As shown in Figure 2b, the CNCs as the solid particles in the aqueous phase exhibited irreversible adsorption at the oil–water interface, and the droplets encapsulating quercetin showed good stability. Due to this, irreversible adsorption occurred at the oil–water interface to form a thick film with high mechanical strength to prevent the agglomeration of droplets, Oswald maturing, and other instability phenomena [39]. With the addition of quercetin, the particle size of the emulation decreased, and the stability of oil droplets increased. In addition, the dense aggregation of oil droplets and solid particles increased the observed particle size of the gel emulsion. The above results were consistent with those of particle size analysis tests. 

### 3.4. Rheological Analysis

**Figure 3:**
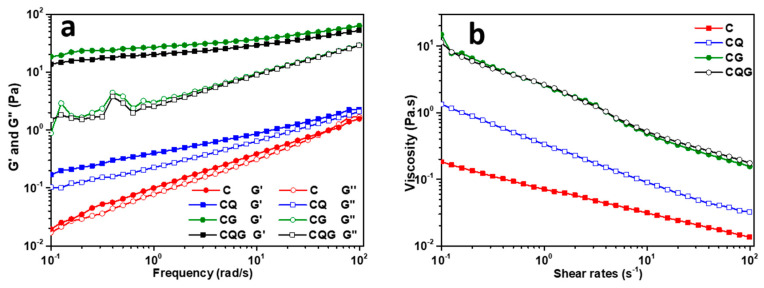
Emulsion rheology diagrams: (**a**) storage modulus G′ and loss modulus G″; (**b**) apparent viscosity as a function of shear rate.

Figure 3a shows the variations in frequency of the storage modulus (G′) and loss modulus (G″) for four types of emulsions. For all samples, G′ > G″, indicating that the emulsions had a well-developed network structure that was elastic and gel-like. In addition, the highest G′ and G″ values were observed in the gel emulsions. Figure 3b shows the variation of the viscosity in the emulsions with cutting rates. Over the tested range of cutting rates, the viscosity of all emulsions decreased with an increasing cutting rate, which was consistent with the properties of non-Newtonian fluids. The cutting–thinning behavior was also observed in an earlier study by Yao et al. [40]. Furthermore, the viscosities of the emulsions differed significantly (*p* < 0.05) at the same cutting rate. The viscosity of the gel emulation was significantly higher than that of the Pickering emulsion, with a corresponding association with the added gelatin solution.

### 3.5. Stability Analysis

**Figure 4:**
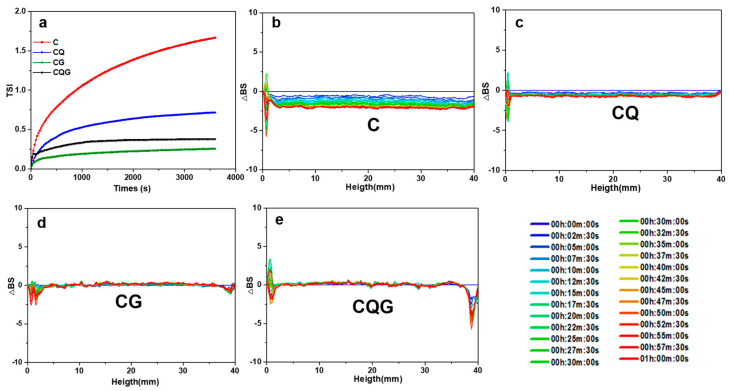
Plot of backscattered luminous flux BS of the emulsion for time and the corresponding stability index (TSI) trend. ((**a**) represents TSI of samples, (**b**–**e**) represent backscattered luminous flux BS of C, CQ, CG, and CQG, respectively).

As shown in Figure 4, the stability of the emulsions was evaluated by multiple dynamic light scattering combined with back-scattered light flow (∆BS) and the stability index (TSI). The variation in ∆BS represented the concentration variation at the top or bottom of the emulsion. The variation in the ∆BS of the emulsion was not significant, indicating the stability of the emulsion. TSI is the sum of all changes in particle size and concentration monitored in the sample volume. The higher the TSI, the less stable the sample is at that time. Obviously, C had the highest TSI value, followed by CQ, indicating that the addition of quercetin could effectively improve the stability of the Pickering emulsion. This result was consistent with the previous observation in the emulsification particle size. The lowest TSI value was observed for the gel emulsion, as the concentration of CNCs affected the size of the formed emulsification droplets. With the increased concentration of CNCs, a denser interfacial layer formed at the interface of the droplet and the stability of the emulsion was further improved [26].

### 3.6. Storage Stability Analysis

**Figure 5:**
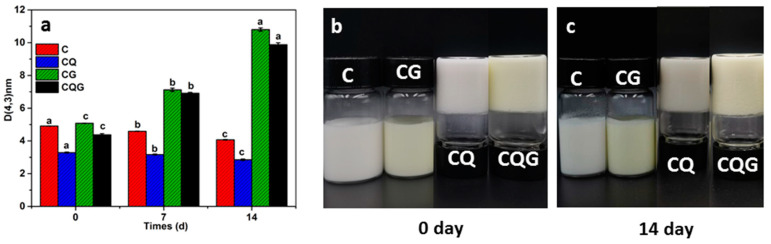
Particle size map (**a**) and macroscopic images of emulsion stored at 4 °C for 0 d (**b**)14 d (**c**). Values with different superscript letters in the same column are significantly different (*p* < 0.05).

The storage stability of the emulsions was evaluated through direct observation after storing at 4 °C for 14 d. As seen in Figure 5, all samples were stable and did not exhibit any phase delamination symptoms. Table 2 shows the particle size and zeta-potential values of the emulsions during storage. The d_[4,3]_ particle size of C gradually decreased from 4.91 to 4.07 μm, while the d_[4,3]_ particle size of CQ decreased from 3.29 to 2.85 μm. This was likely due to the weakening of the core shells of the CNCs on the oil droplets of Pickering emulsions during storage. The average particle size of gel emulsions tended to increase, with the d_[4,3]_ particle size of CG and QG increasing by 112.5% and 126.1%, respectively. The included gelatin likely lessened the electrostatic repulsion of the system and enhanced contact between droplets, causing the flocculation or merging of droplets. Moreover, the system became more unstable when the electrostatic repulsion between droplets decreased [41]. The zeta-potential value is a visual representation of the electrostatic attraction and repellence of emulsions. The absolute value of the zeta potential declined with increasing storage time, and the gel emulsions showed the most obvious declining trend, as shown in Table 2. This finding was in line with the data in the particle size experiments of the emulsion. Overall, the CNCs have enough ability to cover the full droplets quickly and make them resistant.

### 3.7. Oxidative Stability Analysis

**Figure 6:**
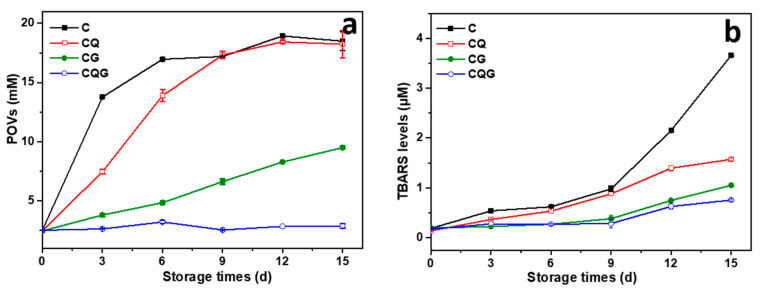
POV values (**a**) and TBARS values (**b**) of emulsions during storage at 50 °C for 15 d.

As presented in Figure 6, all emulsions showed similar changes. The POV and TBAR levels gradually increased. The addition of quercetin reduced the POV and TBAR levels in Pickering emulsions. In addition, lower lipid POV and TBAR were observed in the gel emulsions, indicating the superior oxidative stability of the gel emulsions compared to that of the Pickering emulsion. The gelatin caused the formation of a gel network structure in the emulsions, which led to a smaller oil–water interfacial area and facilitated the prevention of contact between lipid hydroperoxides on the droplet surface and pro-oxidants in the continuous phase [42].

### 3.8. Antioxidant Properties, Encapsulation Rate, and Bioaccessibility Analysis

**Figure 7:**
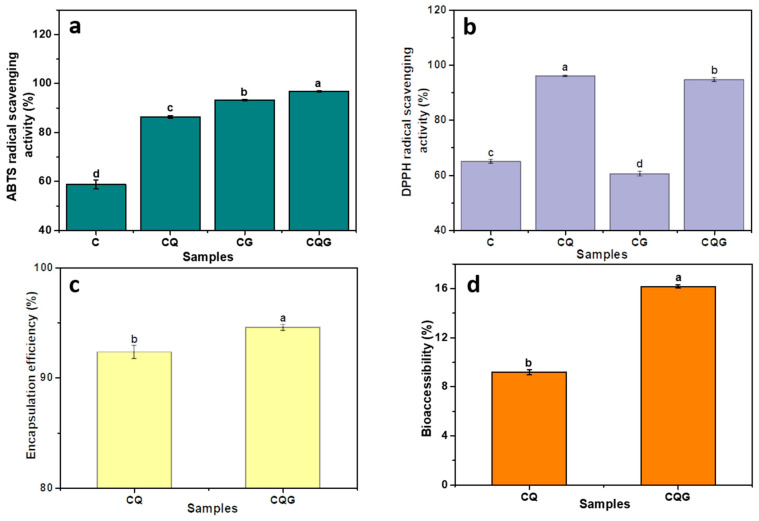
Emulsions for (**a**) ABTS^+^ radical scavenging ability, (**b**) DPPH radical scavenging ability, (**c**) encapsulation rate, and (**d**) bioaccessibility. Values with different superscript letters in the same column are significantly different (*p* < 0.05).

The ABTS^+^ and DPPH radical scavenging capacities are common measurements to evaluate the antioxidant performance of emulsions. As shown in Figure 7, the ABTS^+^ radical scavenging capacities of CQ and CGQ were respectively enhanced by 46.92% and 3.59% with the addition of quercetin compared to those of C and CG. For another, the DPPH radical scavenging capacities of C and CG were 65.08% and 60.59%, respectively, while those of CQ and CGQ were 96.07% and 94.69%, respectively. Hence, the emulsions containing quercetin exhibited more vigorous antioxidant activity than the emulsions without quercetin, mainly due to the potent antioxidant capacity of quercetin. Additionally, the ABTS^+^ radical scavenging ability of the gel emulsion was significantly higher than that of the Pickering emulsification, which indicated that the highly concentrated network structure of the gel emulation could effectively block the movement of free radicals and prevent the entry of oxygen.

The encapsulation rates of CQ and CQG are shown in Figure 7c. CQG had the higher encapsulation rate at 94.57%, while it was 93.4% in CQ. The quercetin in droplets was more tightly encapsulated in the gel network of the gel emulsion than those in the Pickering emulsion. The bioaccessibility for the quercetin-containing emulsions is shown in Figure 7d. In this study, the bioaccessibility of the quercetin in emulsions was ordered as follows: CQG (16.17%) > CQ (9.18%). The higher bioaccessibility of the gel emulsion was related to the dense structure dispersed around the oil droplets, which reduced the degradation of quercetin during digestion [43]. These results suggest the superior drug-delivery ability of gel emulsions compared to Pickering emulsions.

## 4. Conclusions

This work mainly examined the stability and bioaccessibility of quercetin-enriched Pickering emulsion gels stabilized by cellulose nanocrystals extracted from rice bran. All the emulsions presented elastic gel network structures and good stability. Quercetin significantly reduced the particle size of the emulsions and increased their oxidative stability, radical scavenging ability, and antioxidant capacity. Compared to C and CG, the ABTS^+^ radical scavenging capacities of CQ and CQG were enhanced by 46.92% and 3.59%, respectively. In addition, CQG had the higher encapsulation rate at 94.57% and higher bioaccessibility (16.17) compared to CQ. This study indicated that CNCs from rice bran could be exploited as an excellent stabilization particle for a Pickering emulsion and provided a highly stable and bioaccessible delivery system via a Pickering emulsion for water-insoluble functional active factors.

## Figures and Tables

**Table 1:** Emulsion particle size and ζ-potential (*n* = 3).

Samples	d_[4,3]_ (μm)	d_[3,2]_ (μm)	ζ-Potential (mV)
C	4.59 ± 0.01 ^c^	2.93 ± 0.01 ^b^	−41.0 ± 0.15 ^a^
CQ	3.17 ± 0.03 ^d^	2.24 ± 0.01 ^d^	−41.5 ± 0.17 ^a^
CG	7.12 ± 0.10 ^a^	3.15 ± 0.01 ^a^	−12.7 ± 0.29 ^b^
CQG	6.91 ± 0.06 ^b^	2.54 ± 0.01 ^c^	−11.5 ± 0.29 ^c^

Values with different superscript letters in the same column are significantly different (*p* < 0.05).

**Table 2:** Changes in particle size and ζ-potential of emulsions stored at 4 °C for 14 d.

Samples		C	CT	CG	CQG
Day 0	d_[4,3]_ (μm)	4.91 ± 0.01 ^a^	3.29 ± 0.03 ^a^	5.08 ± 0.01 ^c^	4.37 ± 0.08 ^c^
	d_[3,2]_ (μm)	2.87 ± 0.01 ^b^	2.29 ± 0.01 ^a^	2.86 ± 0.00 ^c^	2.41 ± 0.01 ^c^
	ζ-potential (mV)	−42.4 ± 0.21 ^a^	−41.0 ± 0.29 ^b^	−26.8 ± 0.68 ^a^	−27.9 ± 0.26 ^a^
Day 07	d_[4,3]_ (μm)	4.59 ± 0.01 ^b^	3.17 ± 0.03 ^b^	7.12 ± 0.10 ^b^	6.91 ± 0.06 ^b^
	d_[3,2]_ (μm)	2.93 ± 0.01 ^a^	2.24 ± 0.01 ^a^	3.15 ± 0.01 ^b^	2.54 ± 0.01 ^b^
	ζ-potential (mV)	−41.0 ± 0.15 ^b^	−41.5 ± 0.17 ^a^	−25.9 ± 0.06 ^b^	−27.1 ± 0.45 ^a^
Day 14	d_[4,3]_ (μm)	4.07 ± 0.02 ^c^	2.85 ± 0.05 ^c^	10.8 ± 0.10 ^a^	9.88 ± 0.11 ^a^
	d_[3,2]_ (μm)	2.17 ± 0.01 ^c^	1.85 ± 0.01 ^b^	3.33 ± 0.02 ^a^	2.69 ± 0.02 ^a^
	ζ-potential (mV)	−40.0 ± 0.06 ^c^	−40.4 ± 0.26 ^c^	−12.7 ± 0.29 ^c^	−11.5 ± 0.29 ^b^

Values with different superscript letters in the same column are significantly different (*p* < 0.05).

## Data Availability

Data are contained within the article.

## References

[1] Kumar V.D., Verma P.R.P., Singh S.K. (2016). Morphological and in vitro antibacterial efficacy of quercetin loaded nanoparticles against food-borne microorganisms. LWT-Food Sci. Technol..

[2] Kizilbey K. (2019). Optimization of Rutin-Loaded PLGA Nanoparticles Synthesized by Single-Emulsion Solvent Evaporation Method. ACS Omega.

[3] Lei Z., Ning M., Yiliang W., Liyan Z., Gaoxing M., Fei P., Qiuhui H. (2019). Gastrointestinal fate and antioxidation of beta-carotene emulsion prepared by oat protein isolate-Pleurotus ostreatus beta-glucan conjugate. Carbohydr. Polym..

[4] Luo Z.J., Murray B.S., Yusoff A., Morgan M.R.A., Povey M.J.W., Day A.J. (2011). Particle-Stabilizing Effects of Flavonoids at the Oil-Water Interface. J. Agric. Food Chem..

[5] Corrêa A.C., Carmona V.B., Simao J.A., Galvani F., Marconcini J.M., Mattoso L.H.C. (2019). Cellulose Nanocrystals from Fibers of Macauba (*Acrocomia aculeata*) and Gravata (*Bromelia balansae*) from Brazilian Pantanal. Polymers.

[6] Tong Y.Q., Huang S.T., Meng X.J., Wang Y.X. (2023). Aqueous-Cellulose-Solvent-Derived Changes in Cellulose Nanocrystal Structure and Reinforcing Effects. Polymers.

[7] Fatma K., Fedia B., Ramzi K., Araceli G., Julien B., Ellouz C.S. (2016). Isolation and structural characterization of cellulose nanocrystals extracted from garlic straw residues. Ind. Crops Prod..

[8] Goswami A.S., Rawat R., Pillai P., Saw R.K., Joshi D., Mandal A. (2023). Formulation and characterization of nanoemulsions stabilized by nonionic surfactant and their application in enhanced oil recovery. Pet. Sci. Technol..

[9] Cunha A.G., Mougel J.B., Cathala B., Berglund L.A., Capron I. (2014). Preparation of double Pickering emulsions stabilized by chemically tailored nanocelluloses. Langmuir.

[10] Gao H., Ma L., Cheng C., Liu J., Liang R., Zou L., Liu W., McClements D.J. (2021). Review of recent advances in the preparation, properties, and applications of high internal phase emulsions. Trends Food Sci. Technol..

[11] Liu J., Song G., Yuan Y., Zhou L., Wang D., Yuan T., Li L., He G., Yang Q., Xiao G. (2022). Ultrasound-assisted assembly of β-lactoglobulin and chlorogenic acid for non covalent nanocomplex: Fabrication, characterization and potential biological function. Ultrason. Sonochem..

[12] Pan J., Tang L., Dong Q., Li Y., Zhang H. (2021). Effect of oleogelation on physical properties and oxidative stability of camellia oil-based oleogels and oleogel emulsions. Food Res. Int..

[13] Johar N., Ahmad I., Dufresne A. (2012). Extraction, preparation and characterization of cellulose fibres and nanocrystals from rice husk. Ind. Crops Prod..

[14] Kamran S.M., Sadiq B.M., Muhammad A.F., Hafiz K.S. (2014). Rice Bran: A Novel Functional Ingredient. Crit. Rev. Food Sci. Nutr..

[15] Arman S., Hadavi M., Rezvani-Noghani A., Bakhtparvar A., Fotouhi M., Farhang A., Mokaberi P., Taheri R., Chamani J. (2024). Cellulose nanocrystals from celery stalk as quercetin scaffolds: A novel perspective of human holo-transferrin adsorption and digestion behaviours. Luminescence.

[16] Li X.H., Liu Y.Z., Yu Y.Y., Chen W.S., Liu Y.X., Yu H.P. (2019). Nanoformulations of quercetin and cellulose nanofibers as healthcare supplements with sustained antioxidant activity. Carbohydrate. Polymers..

[17] Milutinov J., Krstonosic V., Cirin D., Pavlovic N. (2023). Emulgels: Promising Carrier Systems for Food Ingredients and Drugs. Polymers.

[18] Wang N.Z., Zhang K.D., Chen Y.R., Hu J., Jiang Y.Q., Wang X.B., Ban Q.F. (2023). Tuning whey protein isolate/hyaluronic acid emulsion gel structure to enhance quercetin bioaccessibility and in vitro digestive characteristics. Food Chem..

[19] Chen X., McClements D.J., Wang J., Zou L.Q., Deng S.M., Liu W., Yan C., Zhu Y.Q., Cheng C., Liu C.M. (2018). Coencapsulation of (-)-Epigallocatechin-3-gallate and Quercetin in Particle-Stabilized W/O/W Emulsion Gels: Controlled Release and Bioaccessibility. J. Agric. Food Chem..

[20] Zhang H., Tan S.M., Gan H.M., Zhang H.J., Xia N., Jiang L.W., Ren H.W., Zhang X.A. (2023). Investigation of the formation mechanism and β-carotene encapsulation stability of emulsion gels based on egg yolk granules and sodium alginate. Food Chem..

[21] Song X.Y., Pei Y.Q., Qiao M.W., Ma F.L., Ren H.T., Zhao Q.Z. (2015). Preparation and characterizations of Pickering emulsions stabilized by hydrophobic starch particles. Food Hydrocoll..

[22] Chang S.Q., Chen X., Liu S.W., Wang C. (2020). Novel gel-like Pickering emulsions stabilized solely by hydrophobic starch nanocrystals. Int. J. Biol. Macromol..

[23] Rosa M.F., Medeiros E.S., Malmonge J.A., Gregorski K.S., Wood D.F., Mattoso L.H.C., Glenn G., Orts W.J., Imam S.H. (2010). Cellulose nanowhiskers from coconut husk fibers: Effect of preparation conditions on their thermal and morphological behavior. Carbohydr. Polym..

[24] Li R., He Q., Guo M., Yuan J., Wu Y., Wang S., Rong L., Li J. (2020). Universal and simple method for facile fabrication of sustainable high internal phase emulsions solely using meat protein particles with various pH values. Food Hydrocoll..

[25] Kalita E., Nath B., Deb P., Agan F., Islam R., Saikia K. (2015). High quality fluorescent cellulose nanofibers from endemic rice husk: Isolation and characterization. Carbohydr. Polym..

[26] Sarkar A., Dickinson E. (2020). Sustainable food-grade Pickering emulsions stabilized by plant-based particles. Curr. Opin. Colloid Interface Sci..

[27] Sun L.H., Wang Y.Y., Gong Y.Q. (2022). Life cycle assessment of rice bran oil production: A case study in China. Environ. Sci. Pollut. Res..

[28] Wang S., Yang J., Shao G., Qu D., Zhao H., Yang L., Zhu L., He Y., Liu H., Zhu D. (2020). Soy protein isolated-soy hull polysaccharides stabilized O/W emulsion: Effect of polysaccharides concentration on the storage stability and interfacial rheological properties. Food Hydrocoll..

[29] Thamonwan A., Athikhun S., Sukrit T., Wilailak C., Jirada S. (2017). Fabrication and characterization of rice bran oil-in-water Pickering emulsion stabilized by cellulose nanocrystals. Colloids Surf. A Physicochem. Eng. Asp..

[30] Wang W.Y., Sun C.X., Mao L.K., Ma P.H., Liu F.G., Yang J., Gao Y.X. (2016). The biological activities, chemical stability, metabolism and delivery systems of quercetin: A review. Trends Food Sci. Technol..

[31] Chen W., Ju X., Aluko R.E., Zou Y., Wang Z., Liu M., He R. (2020). Rice bran protein-based nanoemulsion carrier for improving stability and bioavailability of quercetin. Food Hydrocoll..

[32] Qi W., Li T., Zhang Z., Wu T. (2020). Preparation and characterization of oleogel-in-water pickering emulsions stabilized by cellulose nanocrystals—ScienceDirect. Food Hydrocoll..

[33] Xiao J., Lo C., Huang Q.R. (2015). Kafirin Nanoparticle-Stabilized Pickering Emulsions as Oral Delivery Vehicles: Physicochemical Stability and in Vitro Digestion Profile. J. Agric. Food Chem..

[34] Moonchai D., Moryadee N., Poosodsang N. (2012). Comparative properties of natural rubber vulcanisates filled with defatted rice bran, clay and calcium carbonate. Maejo Int. J. Sci. Technol..

[35] Wu X., Li F., Wu W. (2020). Effects of rice bran rancidity on the oxidation and structural characteristics of rice bran protein. LWT.

[36] Zhang X., Qi B., Xie F., Hu M., Sun Y., Han L., Li L., Zhang S., Li Y. (2021). Emulsion stability and dilatational rheological properties of soy/whey protein isolate complexes at the oil-water interface: Influence of pH. Food Hydrocoll..

[37] Xu N., Wu X.L., Zhu Y.Q., Miao J.Y., Gao Y., Cheng C., Peng S.F., Zou L.Q., McClements D.J., Liu W. (2021). Enhancing the oxidative stability of algal oil emulsions by adding sweet orange oil: Effect of essential oil concentration. Food Chem..

[38] Xu Q.Q., Qi B.K., Han L., Wang D.Q., Zhang S., Jiang L.Z., Xie F.Y., Li Y. (2021). Study on the gel properties, interactions, and pH stability of pea protein isolate emulsion gels as influenced by inulin. LWT Food Sci. Technol..

[39] Aditya N., Macedo A.S., Doktorovova S., Souto E.B., Kim S., Chang P.-S., Ko S. (2014). Development and evaluation of lipid nanocarriers for quercetin delivery: A comparative study of solid lipid nanoparticles (SLN), nanostructured lipid carriers (NLC), and lipid nanoemulsions (LNE). LWT Food Sci. Technol..

[40] Yao X., Cao J., Teng W., Li J., Wang J. (2023). Effects of W/O Nanoemulsion on Improving the Color Tone of Beijing Roast Duck. Foods.

[41] Xiao Y., Liu Y., Wang X., Li M., Lei H., Xu H. (2019). Cellulose nanocrystals prepared from wheat bran: Characterization and cytotoxicity assessment. Int. J. Biol. Macromol..

[42] Wu Y., Chen F., Zhang C., Lu W., Gao Z., Xu L., Wang R., Nishinari K. (2021). Improve the physical and oxidative stability of O/W emulsions by moderate solidification of the oil phase by stearic acid. LWT.

[43] Zhao G.H., Zhang R.F., Dong L.H., Huang F., Tang X.J., Wei Z.C., Zhang M.W. (2018). Particle size of insoluble dietary fiber from rice bran affects its phenolic profile, bioaccessibility and functional properties. LWT Food Sci. Technol..

